# Enhanced the Trans‐Cleavage Activity of CRISPR‐Cas12a Using Metal‐Organic Frameworks as Stimulants for Efficient Electrochemical Sensing of Circulating Tumor DNA

**DOI:** 10.1002/advs.202417206

**Published:** 2025-04-04

**Authors:** Shuai Wu, Yincheng Liu, Tianyu Zeng, Tianci Zhou, Yanting Sun, Ying Deng, Juan Zhang, Genxi Li, Yongmei Yin

**Affiliations:** ^1^ Clinical Research Center The First Affiliated Hospital with Nanjing Medical University Nanjing Jiangsu 210029 P. R. China; ^2^ Department of Breast Disease The First Affiliated Hospital of Nanjing Medical University Nanjing 210029 P. R. China; ^3^ Department of Oncology The First Affiliated Hospital of Nanjing Medical University Nanjing 210029 P. R. China; ^4^ State Key Laboratory of Analytical Chemistry for Life Science School of Life Sciences Nanjing University Nanjing 210023 P. R. China; ^5^ Center for Molecular Recognition and Biosensing School of Life Sciences Shanghai University Shanghai 200444 P. R. China

**Keywords:** Cas12a/crRNA, circulating tumor DNA, CRISPR‐based diagnostics, electrochemical biosensor, metal‐organic framework

## Abstract

Continued development of clustered regularly interspaced short palindromic repeats (CRISPR)‐powered biosensing system on the electrochemical interface is vital for accurate and timely diagnosis in clinical practice. Herein, an electrochemical biosensor based on manganese metal‐organic frameworks (MOFs)‐enhanced CRISPR (MME‐CRISPR) is proposed that enables the efficient detection of circulating tumor DNA (ctDNA). In this design, customized enzyme stimulants (Mn^2+^) are co‐assembled with Cas12a/crRNA to form enzyme‐MOF composites, which can be released quickly under mild conditions. The MOFs‐induced proximity effect can continuously provide adequate Mn^2+^ to sufficiently interact with Cas12a/crRNA during the release process, enhancing the trans‐cleavage activity of complex available for biosensor construction. The MOFs‐based enzyme biocomposites also afford efficient protection against various external stimulus. It is demonstrated that the developed biosensor can achieve ultrasensitive detection of epidermal growth factor receptor L858R mutation in ctDNA with a low detection limit of 0.28 fm without pre‐amplification. Furthermore, the engineered mismatch crRNA enables the biosensor based on MME‐CRISPR to detect single nucleotide variant with a high signal‐to‐noise ratio. More importantly, it has been successfully used to detect the targets in clinical practice, requiring low‐dose samples and a short time. This strategy is believed to shed new light on the applications of cancer diagnosis, treatment, and surveillance.

## Graphical abstract

This work designs manganese metal‐organic frameworks (Mn‐MOFs) co‐assembled with Cas12a/crRNA, enabling rapid release of Mn^2^⁺ under mild conditions. The MOFs‐induced proximity effect sustains Mn^2^⁺‐Cas12a/crRNA interactions, enhancing the trans‐cleavage activity of complex available to develop an electrochemical biosensor based on Mn‐MOFs‐enhanced clustered regularly interspaced short palindromic repeats   system, which offers a scalable platform for the ultrasensitive detection of circulating tumor DNA mutation.

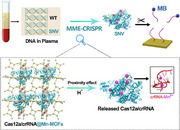

## 1 Introduction

Accurate, rapid, and portable sensing of nucleic acid with high sensitivity will make a difference in numerous clinical diagnostic,^[^ ^1^ ^]^ especially by precisely profiling and monitoring circulating tumor DNA (ctDNA) to direct therapeutic management in targeted cancer therapy.^[^ ^2^, ^3^ ^]^ However, current nucleic acid amplification‐based methods and sequencing techniques remain great challenges associated with the identification for the extremely low abundance of somatic mutation hotspots in liquid biopsies.^[^ ^4^ ^]^ Intriguingly, clustered regularly interspaced short palindromic repeats (CRISPR) system,^[^ ^5^ ^]^ a powerful gene editing technique, has recently been extended to develop novel biosensing strategies for nucleic acid detection due to their excellent target recognition ability, programmability, and reliability.^[^ ^6^ ^]^ The key attribute of CRISPR‐based biosensing is the unique trans‐cleavage activity of certain RNA‐guided CRISPR‐associated (Cas) nucleases, such as Cas12a, Cas13a, and Cas14, toward single‐stranded oligonucleotides.^[^ ^7^ ^]^ By programming the Cas/crRNA to recognize target substrate, varieties of methods have been proposed, such as Specific High Sensitivity Enzymatic Reporter Unlocking^[^ ^8^ ^]^ or DNA Endonuclease‐Targeted CRISPR Trans Reporter system.^[^ ^9^ ^]^ In view of the lack of sensitivity of these CRISPR‐based assays in earlier studies, particularly relying on pre‐amplification procedure of the target sequence,^[^ ^10^, ^11^ ^]^ much effort has been contributed to manipulate the enzymatic kinetic reaction, including protein engineering strategies,^[^ ^12^, ^13^ ^]^ controlling of regulatory crRNA structure,^[^ ^14^, ^15^ ^]^ and changing the output of the signal probes.^[^ ^16^, ^17^ ^]^ In regard of these studies to improve the trans‐cleavage activity, it can be found that whether using protein variants or engineering crRNA, or combining signal probes with different nanostructures,^[^ ^18^, ^19^ ^]^ the characteristics of natural CRISPR‐Cas nucleases that are easily inactivated in harsh environments such as elevated temperature, the co‐existences of inhibitors and most organic solvents, are not taken into account, which undoubtedly limits their further diagnostic applications. Therefore, improving enzyme performance by increasing enzyme activity while ensuring stability will be of great significance and urgently desired for the practical applications of ctDNA detection.

Encapsulating the enzyme in a matrix such as a cross‐linked network or bulk polymer can well regulate the physical environment around the enzyme.^[^ ^20^ ^]^ It is a very effective way to improve enzyme stability, but also tend to reduce the ability of the substrate to diffuse to the enzyme, thereby inhibiting its activity. In particular, the substrate of CRISPR‐Cas nucleases is a single‐stranded or double‐stranded nucleic acid chain, which is difficult to diffuse through the pores of the matrix to interact with enzyme. Therefore, we speculate that if an immobilized carrier can grow in a controllable manner on the surface of the CRISPR‐Cas nucleases to equip them with a layer of armor to protect the enzyme activity, and can also provide activators to increase the enzyme activity when they are needed, it will effectively address the two concerns of sensitivity and stability, facilitating the accurate detection of ctDNA.

Metal‐organic frameworks (MOFs), which are periodic 3D network materials connected by metal‐containing units and multitopic organic linker,^[^ ^21^ ^]^ have been gaining popularity for the preparation and application of MOF‐enzyme composites in recent years.^[^ ^22^, ^23^ ^]^ Due to their modular construction and balanced flexibility,^[^ ^24^, ^25^ ^]^ the tunable cavities and tailorable chemistry of MOFs can be precisely adjusted to encapsulate a specific enzyme via a de novo approach such as coprecipitation or biomimetic mineralization, enabling better performance as compared to free enzymes.^[^ ^26^, ^27^ ^]^ Additionally, the self‐assembly behavior at the bio‐interface between MOFs and enzymes can be optimized to control efficient formation or degradation of the framework under mild conditions.^[^ ^28^, ^29^ ^]^ That is to say, MOFs may be a powerful class of stimulants, which can interact with CRISPR‐Cas nucleases to enhance the trans‐cleavage activity for nucleic acid sensing.

**Scheme 1:**
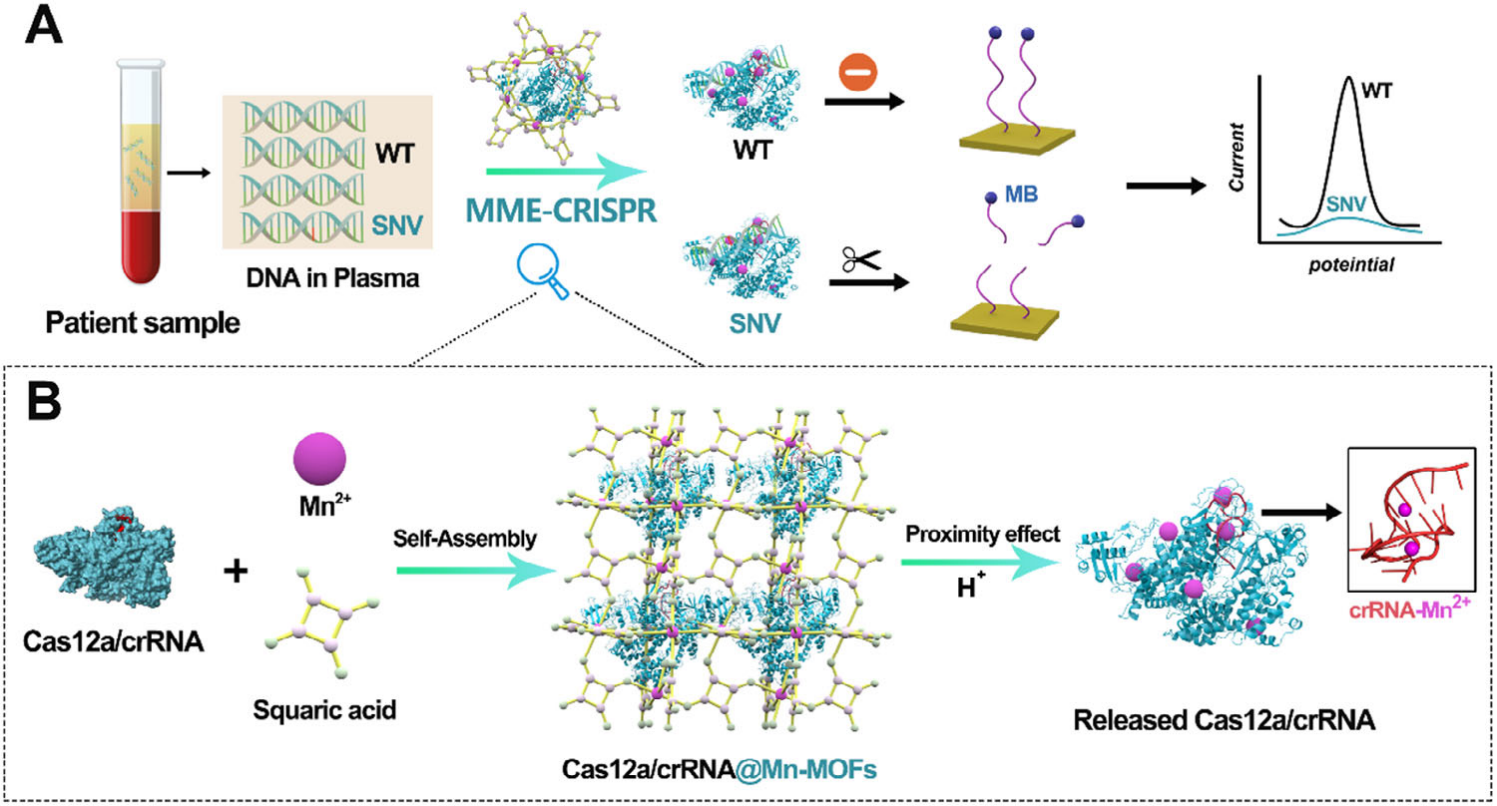
The principle and workflow of the enhanced electrochemical biosensor based on MME‐CRISPR. A) Typical workflow of released Cas12a/crRNA with enhanced activity for efficient electrochemical DNA sensing in patient samples. B) Schematic diagram for the crystallization of Cas12a/crRNA@Mn‐MOFs and the degradation of Mn‐MOFs to release Cas12a/crRNA under mild conditions.

In this work, we have reported an efficient and biocompatible strategy that enables controllable integration of manganese metal‐organic frameworks (Mn‐MOFs) and CRISPR‐Cas nucleases to greatly enhance the catalytic activity, named as MME‐CRISPR, which can be used for constructing an electrochemical biosensor for ultrasensitive detection of ctDNA. The principle and workflow are shown in **Scheme** **1**A, there is a large amount of wild‐type (WT) cell‐free DNA and low abundance of mutant ctDNA, especially the single nucleotide variant (SNV), in the plasma samples obtained from cancer patients. To address this challenge, MME‐CRISPR is introduced into the electrochemical interface to achieve accurate determination. The MME‐CRISPR design is shown in Scheme 1B, MOFs composed of Mn^2+^ are co‐assembled with Cas12a/crRNA duplex to form enzyme‐MOF composites under ambient conditions. The generated Mn‐MOF shells can interact tightly around the Cas12a proteins via the efficient self‐organization approach. It should be noted that framework stabilization can shelter the duplex from environmental stressors. More interestingly, we found that the Mn^2+^ can be released quickly from the framework, serving as the stimulants to interact with Cas12a/crRNA duplex. The sufficient interaction medicated by the MOFs‐induced proximity effect can greatly enhance the trans‐cleavage activity. Thus, the substrate single‐stranded DNA (ssDNA) labeled with methylene blue (MB‐DNA) on the electrode can be efficiently cleaved to generate strong signal. As a result, with the excellent target recognition of Cas12a/crRNA duplex, target dsDNA can be detected with high accuracy and sensitivity using the developed biosensor based on MME‐CRISPR. Moreover, this strategy shows great performance in analysis of clinical samples, which may provide an alternative tool for CRISPR‐based diagnosis.

## 2 Results and Discussion

### 2.1 Preparation and Characterization of Cas12a/crRNA@Mn‐MOFs

As known to all, divalent metal ions coordinated in the RuvC endonuclease domains of Cas12a proteins, particularly magnesium ions (Mg^2+^), are well‐documented as indispensable cofactors for the enzymatic activity.^[^ ^30^ ^]^ However, there are few reports on the synthesis of Mg‐based MOFs under mild conditions. Inversely, manganese ions (Mn^2+^), as another kind of efficient activator for Cas12a, have been utilized to explore MOFs.^[^ ^31^, ^32^ ^]^ Systematically, a comparative assays with other divalent metal ions such as zinc ion (Zn^2^⁺), ferrous ion (Fe^2+^), calcium ion (Ca^2+^), and copper ion (Cu^2^⁺) have been conducted using FAM/BHQ‐labeled single‐stranded DNA (FQ‐DNA), it can be found that Mn^2^⁺ confers the highest activation efficiency of Cas12a/crRNA in the presence of different concentration target dsDNA at pH 7.4 (Figure S1, Supporting Information). We also found that there is no significant difference in its effect on the activity of Cas12a protein between the Mn^2+^ group and a combination of two metal ions using Mn^2+^/Mg^2+^. These results make Mn^2^⁺ the most balanced choice. However, the CRISPR‐Cas12a subtype used in the above experiments is LbCas12a. To assess the generalizability of Mn^2^⁺‐induced enhancement, we have tested three additional Cas12 family members including AsCas12a, FnCas12a, and AacCas12b. As shown in Figure S2A Supporting Information(), Mn^2+^ can enhance the activity of all these proteins, with the best activity enhancement observed in AsCas12a. However, its specificity was relatively low (Figure S2B, Supporting Information), which is consistent with previous work.^[^ ^33^ ^]^ Therefore, LbCas12a has been chosen for the research.

**Figure 1:**
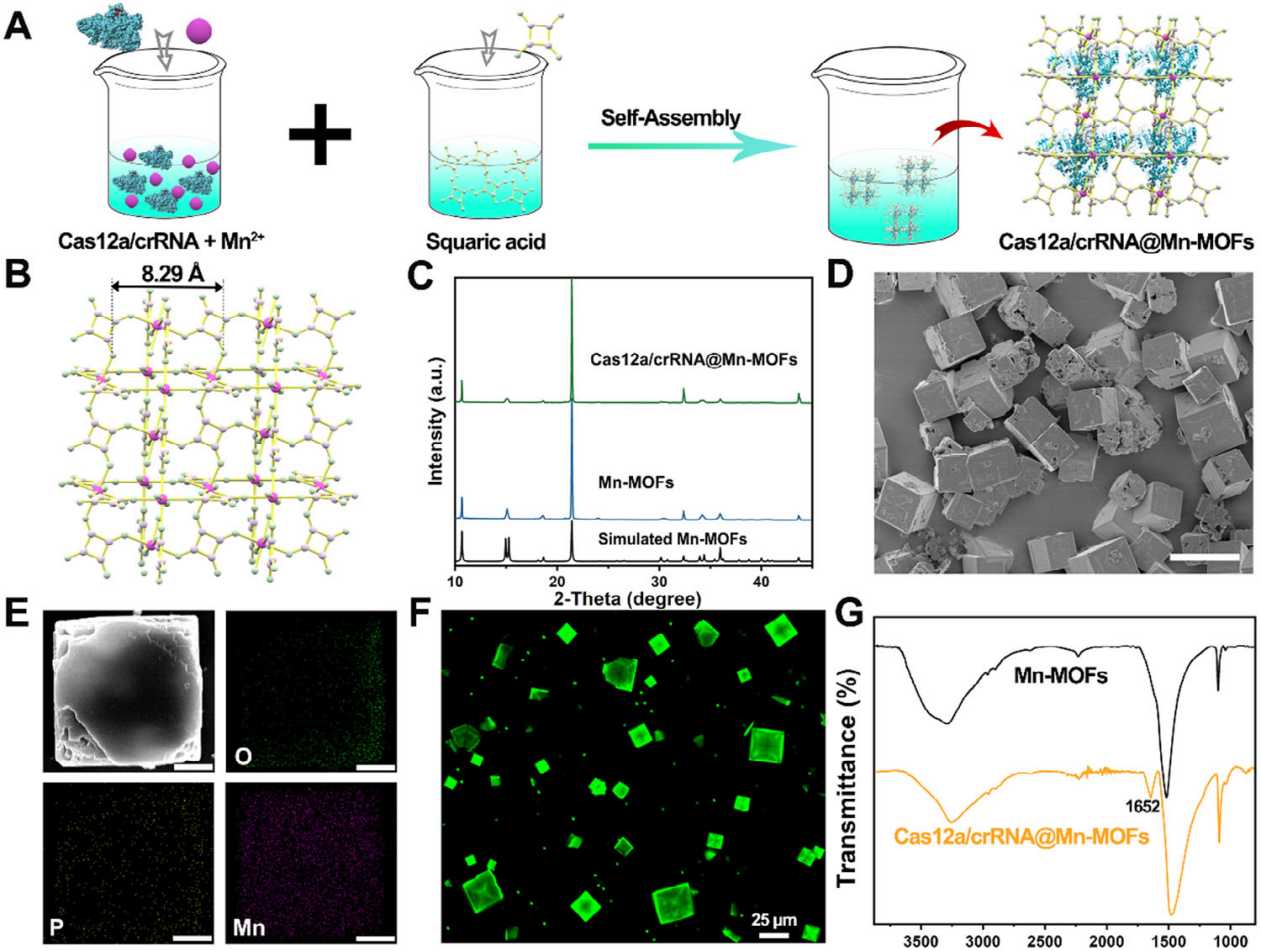
Characterization of Cas12a/crRNA@Mn‐MOFs. A) Schematic illustration for the preparation process of Cas12a/crRNA@Mn‐MOFs. B) Simulated crystal structure of Mn‐MOFs. C) X‐ray diffraction graphs of simulated Mn‐MOFs, actual Mn‐MOFs, and Cas12a/crRNA@Mn‐MOFs. D) Scanning electron microscopic micrographs of Cas12a/crRNA@Mn‐MOFs, the scale bar is 20 µm. E) The corresponding elemental mapping of the Cas12a/crRNA@Mn‐MOFs, the scale bar is 5 µm. F) Confocal microscopic images of Cas12a/FAM‐crRNA@Mn‐MOFs. G) FT‐IR spectra of Mn‐MOFs and Cas12a/crRNA@Mn‐MOFs.

In our work, Cas12a/crRNA@Mn‐MOFs are innovatively designed via a *de novo* approach, in which Cas12a/crRNA was pre‐mixed with Mn^2+^ solution, followed by the addition of squaric sodium solution under stirring (**Figure** **1**A). First, the crystal structure of Mn‐MOFs is simulated in Figure 1B. Then, the prepared Cas12a/crRNA@Mn‐MOFs has been examined by X‐ray diffraction (XRD) (Figure 1C), the observed sharp and high‐intensity peaks in the pattern suggest the presence of crystal material, major diffraction peaks at 2θ values of 10.6°, 15.2°, 18.5°, 21.4°, and 32.1° are corresponded to the crystal data reported in the previous literature.^[^ ^34^ ^]^ The results well confirm their sodalite structural analogy to prototypical Mn‐MOFs with good purity. Scanning electron microscope (SEM) image reveals that Cas12a/crRNA and Mn‐MOFs are co‐assembled to form microcrystals (Figure 1D). To assess spatial distribution of Cas12a/crRNA in the frame structure, the energy dispersive X‐ray spectroscopy (EDS) mapping has been conducted. As shown in Figure 1E, the oxygen (O) element and the phosphorus (P) derived from the Cas12a/crRNA, as well as Mn element distributed throughout the crystals, indicating the successful encapsulation of Cas12a/crRNA in the cavity of Mn‐MOFs. To determine the spatial distribution of Cas12a/crRNA within Mn‐MOFs, fluorescein (FAM) has been labeled at 3′ end of crRNA to prepare Cas12a/FAM‐crRNA@Mn‐MOFs, which are used for analysis by confocal laser scanning microscopy (CLSM). As shown in Figure 1F, the fluorescence of FAM can be found within the Mn‐MOFs, further demonstrating the successful encapsulation of Cas12a/crRNA. Additionally, Fourier transform infrared spectroscopy (FT‐IR) analysis shows that a characteristic band at 1652 cm^−1^ ascribed to the stretching vibration of C═O group of peptide bond appears in comparison with that of pure Mn‐MOFs (Figure 1G). These results well verify the successful synthesis of Cas12a/crRNA@Mn‐MOFs.

### 2.2 Release of Cas12a/crRNA from Mn‐MOFs and Its Catalytic Efficiency and Stability

**Figure 2:**
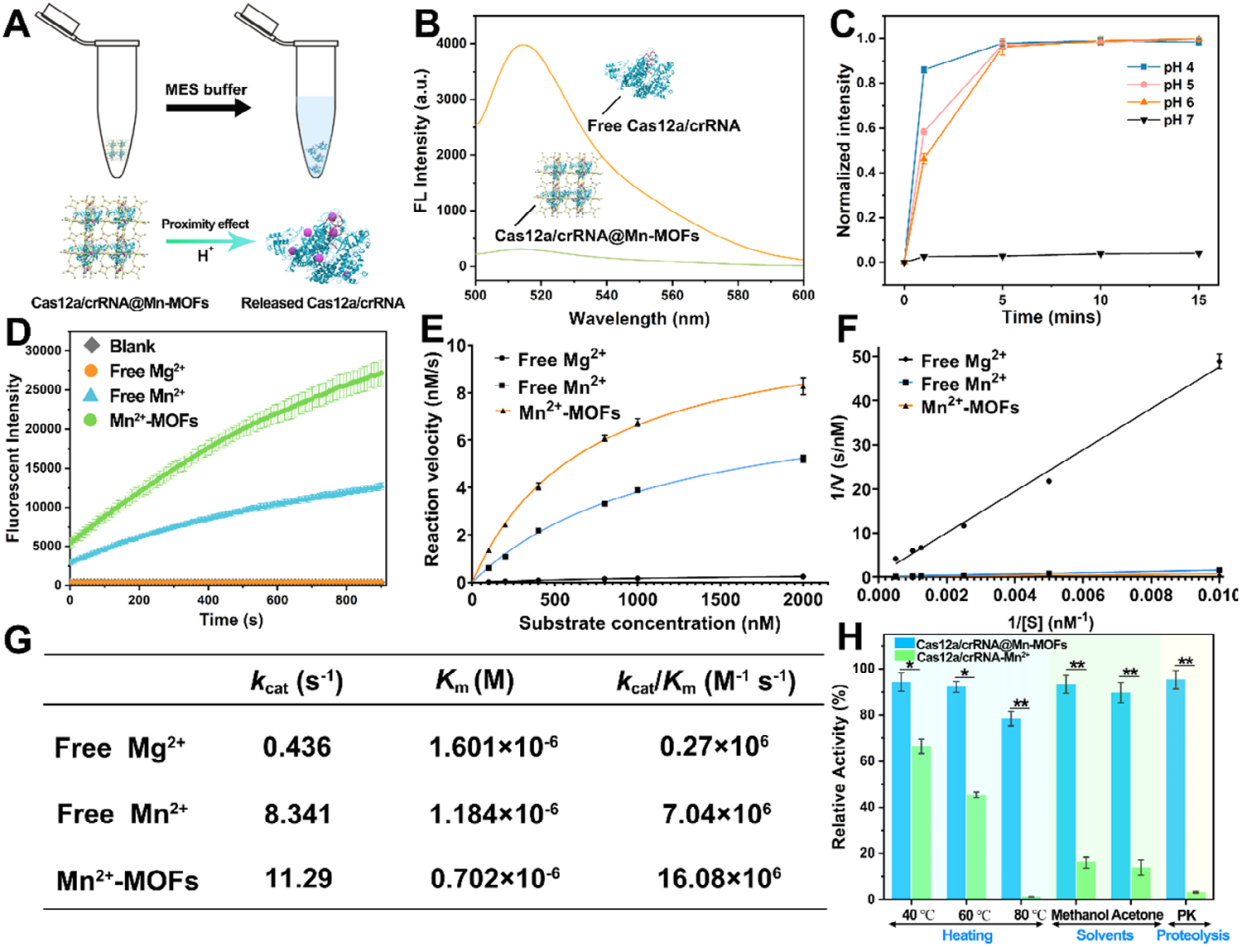
Release of Cas12a/crRNA from Mn‐MOFs and its catalytic efficiency and stability. A) Schematic illustration for release of Cas12a/crRNA from Mn‐MOFs. B) Fluorescent spectra of the supernatant from Cas12a/crRNA@Mn‐MOFs compared to free Cas12a/crRNA duplex. C) Release of Cas12a/crRNA from Mn‐MOFs with different pH values. D) The changes of fluorescence intensities along with the reaction time obtained for free Cas12a/crRNA with Mg^2+^ or Mn^2+^ at pH 7.4, and Cas12a/crRNA@Mn‐MOFs at pH 6 in the presence of 0.1 nm target dsDNA. E) Michaelis‐Menten plot for the cleavage activity of free Cas12a/crRNA with Mg^2+^ and Mn^2+^, and Cas12a/crRNA@Mn‐MOFs. F) Corresponding Lineweaver‐Burk plot for the cleavage activity of free Cas12a/crRNA with Mg^2+^ and Mn^2+^, and Cas12a/crRNA@Mn‐MOFs. G) Enzyme kinetic parameters of catalytic reaction. H) Stability for free Cas12a/crRNA with Mn^2+^ and Cas12a/crRNA@Mn‐MOFs. Student's t test shows statistical significance: *p < 0.05 and **p < 0.01. Error bars represent standard derivation obtained in three parallel experiments (*n* = 3).

As schemed in **Figure** **2**A, Cas12a/crRNA can be released from Mn‐MOFs under appropriate condition. First, loading capacity is evaluated by using FAM‐crRNA to form Cas12a/FAM‐crRNA to record the change of fluorescence intensity at 515 nm. As shown in Figure 2B, the fluorescence intensity of the supernatant solution after the assembly is significantly lower than that of the free Cas12a/crRNA, exhibiting the high guest encapsulation capacity. The loading efficiency can be calculated to be 92.4% according to the established standard curve (Figure S3, Supporting Information). The frame around Cas12a/crRNA can prevent the approach of target dsDNA. As shown in Figure 2C, a very low signal can be found at pH 7, which can be explained for the prevention effect of frame crystal structure on the Cas12a/crRNA.

On contrary, the high signal appears under acidic conditions (pH 4, 5, and 6), which well verifies the efficient release of Cas12a/crRNA from frame structure. Considering the impact of pH on activity, a pH of 6 has been chosen as the experimental condition for the release of Cas12a/crRNA.

Catalytic efficiency of Cas12a/crRNA@Mn‐MOFs has been further evaluated. First, Cas12a/crRNA in the commercial buffer (1 × NEBuffer r2.1 containing 10 mm Mg^2+^) and FQ‐DNA were mixed with different concentrations of target dsDNA. As shown in Figure S4 (Supporting Information), the fluorescence signal gradually increases with the increased concentrations of target dsDNA, demonstrating that the effective activation of Cas12a by target dsDNA. However, when the target DNA concentration is very low such as 0.1 nm, the signal consistent with the background can only be observed in the free Cas12a/crRNA system with free Mg^2+^ (Figure 2D), suggesting that Cas12a is not activated. Compared with those for free Cas12a/crRNA with free Mg^2+^ or Mn^2+^ at pH 7.4, the evidently high fluorescence can be observed for Cas12a/crRNA@Mn‐MOFs. It can be found that the time‐fluorescent intensity curve of Mn^2+^‐MOFs‐medicated assay (pH 6) is stronger than that of free Mn^2+^ (pH 7.4) in the presence of 0.1 nm target dsDNA, exhibiting a markedly enhanced enzyme activity. Certainly, the activity of Cas12a/crRNA using free Mn^2+^ at pH 6 is further reduced (Figure S5, Supporting Information). Moreover, in order to calculate *k*_cat_ and *K*_m_ values, a linear correlation between the fluorescence intensity and the concentration of the completely cleaved reporter substrate has been first established (Figure S6, Supporting Information). Then, the kinetic analysis of Cas12a trans‐cleavage activity under different experimental conditions was measured in Figures S7–S9 (Supporting Information). As shown in Figure 2E,F, an obviously increased reaction rate can be observed for Cas12a/crRNA@Mn‐MOFs, in comparison with those for free Cas12a/crRNA with Mn^2+^ or Mg^2+^. Clearer graphs for the corresponding Michaelis‐Menten plot and Lineweaver‐Burk plot are shown in Figures S10 and S11 (Supporting Information). Meanwhile, a 59‐fold increase in *k*_cat_/*K*_m_ values can be found for Cas12a/crRNA@Mn‐MOFs (16.08 × 10^6^ M^−1^ s^−1^) than that for free Cas12a/crRNA with Mg^2+^ (0.27 × 10^6^ M^−1^ s^−1^) (Figure 2G). Although the pH is 6, the activity of the Cas12a/crRNA@Mn‐MOFs is still much higher than that of the free Cas12a/crRNA system at pH 7.4. These results well confirm the tremendous catalytic ability of Cas12a/crRNA@Mn‐MOFs.

To reveal the specific mechanism for the enhanced trans‐cleavage activity of CRISPR‐Cas12a, we have investigated the release kinetics of Mn^2+^ ions using inductively coupled plasma optical emission spectroscopy (ICP‐OES) to determine the concentration of Mn^2+^ bound to the released Cas12a/crRNA after ultrafiltration at different time. As shown in Figure S12 (Supporting Information), the results show that Cas12a/crRNA could hold high concentrations of Mn^2+^ for a certain period of time after the material degradation. The presence of large amounts of Mn^2+^ binding with crRNA can accelerate the protein conformational change with the formation of R‐loop structure, thereby significantly promoting the binding and cleavage of RuvC endonuclease domains with the substrate ssDNA and reducing the value of *K*m.^[^ ^35^, ^36^ ^]^ It can be concluded that Mn‐MOFs provide a large amount of metal ions to serve as the stimulants to interact with Cas12a/crRNA duplex and MOFs‐induced proximity effect can greatly enhance the trans‐cleavage activity.

Furthermore, we have further evaluated the protective effect of Mn‐MOFs on Cas12a/crRNA toward external perturbation such as heating, organic solvents, and proteolysis. Thermal stabilities of Cas12a/crRNA@Mn‐MOFs and free Cas12a/crRNA are assessed by dealing with high temperatures at 40, 60, and 80 °C. As shown in Figure 2H, the relative activity of free Cas12a/crRNA with Mn^2+^, which is normalized to the original activity of Cas12a/crRNA before treatment, decreases greatly after treatment with elevated temperature, especially at 80 °C. However, Cas12a/crRNA@Mn‐MOFs retains over 90% bioactivity at 40 or 60 °C and ≈80% bioactivity at 80 °C, exhibiting a high thermal stability. Additionally, the organic solvent tolerance has been tested by exposing samples to methanol and acetone for 1 h. As expected, Cas12a/crRNA@Mn‐MOFs show better chemical resistance than free Cas12a/crRNA with Mn^2+^. Notably, the MOFs‐protected Cas12a/crRNA effectively resists degradation by proteolytic enzymes (Proteinase K, PK), whereas the free Cas12a/crRNA system can barely detect targets. Moreover, to further evaluate their long‐term storage capacity, the two systems were kept at room temperature for two weeks. As shown in Figure S13 (Supporting Information), Cas12a/crRNA@Mn‐MOFs can maintain high activity for a longer period, while the free system quickly loses its activity next day. Collectively, these results demonstrate that the fabrication of shell skeleton using Mn‐MOFs have an extraordinary protective effect on the CRISPR‐Cas12a system.

### 2.3 Establishment of Electrochemical Method Based on MME‐CRISPR

**Figure 3:**
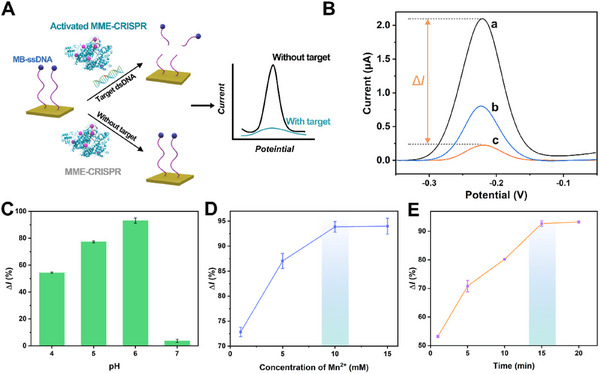
Establishment of electrochemical method based on Cas12a/crRNA@Mn‐MOFs. A) Schematic illustration for the established electrochemical method. B) Square wave voltammograms of MB‐DNA modified gold electrodes. (a) Cas12a/crRNA@Mn‐MOFs without target dsDNA, (b) free Cas12a/crRNA + 10 mm Mn^2+^ + target dsDNA, and (c) Cas12a/crRNA@Mn‐MOFs + target dsDNA. Δ*I* (%) = (Background current – current with target dsDNA) / Background current × 100. The change of current obtained by using (C) MES buffer with different pH values, D) different concentrations of Mn^2+^ for the preparation of Cas12a/crRNA@Mn‐MOFs, and E) different incubation time on the interface of the modified electrodes. Error bars represent standard derivation obtained in three parallel experiments (*n* = 3).

The electrochemical biosensor has been established based on MME‐CRISPR. As schemed in **Figure** **3**A, MB‐DNA has been modified on the surface of gold electrode as signal probes. The target dsDNA is mixed with Cas12a/crRNA@Mn‐MOFs in acidic buffer (pH 6), followed by incubation on the prepared electrochemical interface. The released MME‐CRISPR activated by target dsDNA can cleave MB‐DNA on the gold electrode, resulting in the departure of MB from the interface and the correspondingly decreased electrochemical signal.

First, electrochemical impedance spectroscopy (EIS) has been utilized to evaluate the stepwise modification process of the working electrode. As shown in Figure S14 (Supporting Information), the bare gold electrode (curve a) exhibits an extremely low electron transfer resistance (*R*_et_). After the modification with MB‐DNA, an increasing diameter of semicircle can be observed in curve b. Then the activated MME‐CRISPR is introduced into the electrochemical interface, the *R*_et_ value decreases significantly (curve c), implying the successful progress of cleavage reaction. Additionally, square wave voltammograms (SWV) measurements have been further used to evaluate the feasibility of the biosensor based on MME‐CRISPR. As shown in Figure 3B, a high signal can be found without target dsDNA (curve a), as a result of un‐activation of Cas12a and the remaining of MB on the surface of electrode. In the presence of target dsDNA (0.1 nm), the distinct decreased current appears for Cas12a/crRNA@Mn‐MOFs (curve c), implying the cleavage of a large amount of MB‐DNA from the electrode surface. Moreover, the peak current values are evidently low than that for free Cas12a/crRNA with Mn^2+^ (10 mm). These results well demonstrate that Cas12a/crRNA released from Mn‐MOFs can achieve an improved enzymatic activity, resulting from the proximity effect of Mn^2+^ in frame structure.

The pH values for the release of Cas12a/crRNA from Mn‐MOFs have been further investigated. As shown in Figure 3C, nearly no current change occurs with the addition of target dsDNA into Cas12a/crRNA@Mn‐MOFs in MES buffer at pH 7. It can be explained for integrity of the frame crystal structure which inhibits the binding of target dsDNA with Cas12a/crRNA. Inversely, the clear current change can be found under acidic conditions, verifying the efficient release of Cas12a/crRNA from Mn‐MOFs and its subsequent binding with target dsDNA. With the aid of Mn^2+^ in the frame structure, the activated Cas12a can effectively cleave the DNA strand on the surface of the modified electrode, resulting in the departure of MB and the corresponding decrease of electrochemical current signal. Certainly, the reduced current appears along with the decrease of pH values of MES buffer, which is consistent with widespread knowledge that enzyme activity reduces under acidic conditions. Meanwhile, to further investigate the effects of pH on the methylene blue redox signal due to Cas12a cleavage, the SWV curves of MB‐DNA on electrode have been recorded with different pH values (pH 4, 5, and 6). As shown in Figure S15 (Supporting Information), it can be found that the peak position has a right shift with the decrease of pH values, but the change of current recorded after the cleavage reaction is not significant. Therefore, pH 6 has been selected to be the optimized value for this method. In addition, the concentration of Mn^2+^ used for constructing MOFs is a key to enhance the enzyme activity. As clearly shown in Figure 3D, the current change reaches a plateau at the concentration of 10 mm, providing the appropriate amount of metal ions to stimulate the catalytic ability of activated Cas12a. Otherwise, the cleavage process will be accomplished within 15 min (Figure 3E).

### 2.4 Design of crRNA for the Detection of Circulating Tumor DNA

**Figure 4:**
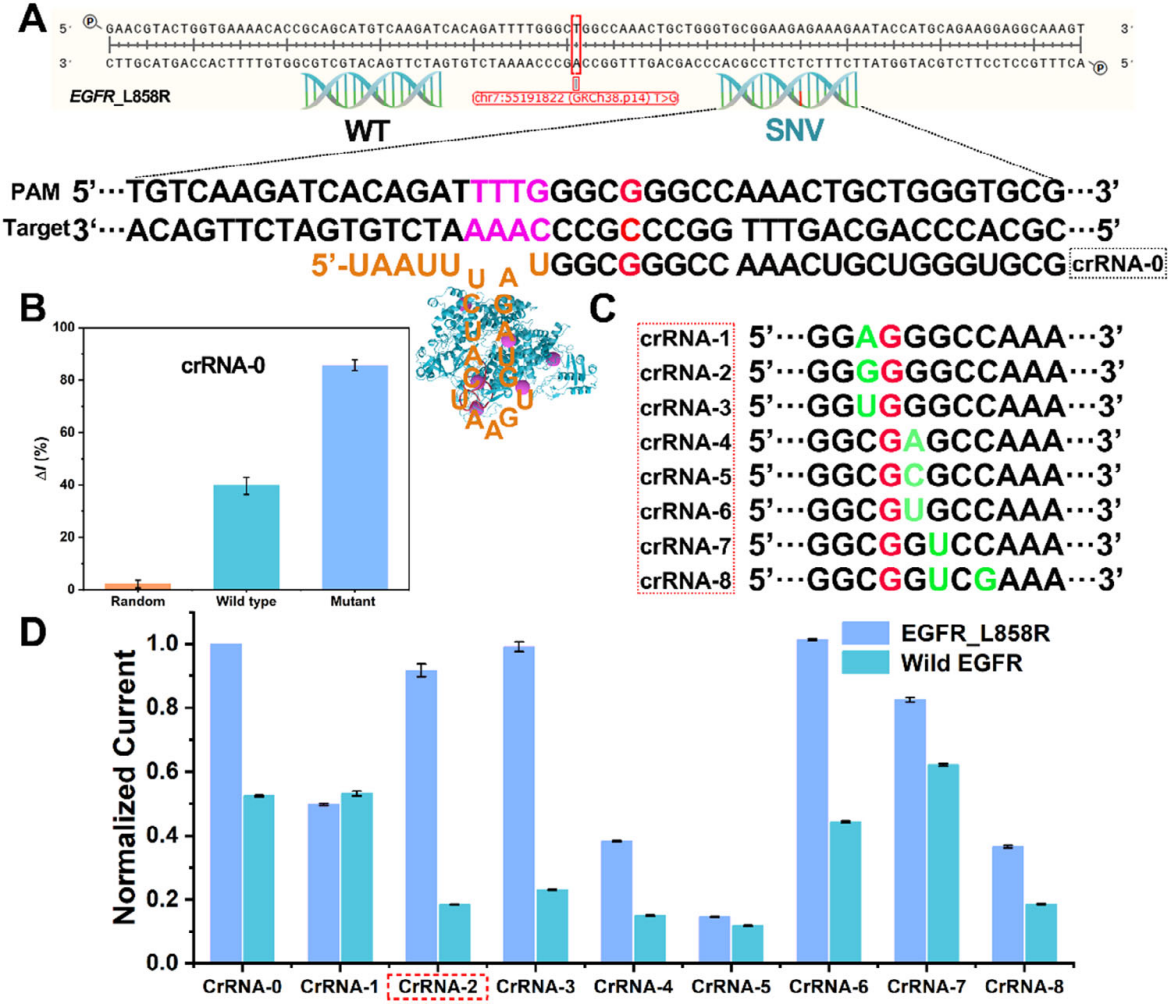
Design of crRNA for the detection of ctDNA. A) Schematic of the base mutation between the wild type and mutant (A‐to‐C) in the fragment of gene *EGFR* for the design of crRNA sequence based on the requirements of PAM sequence (‐TTTN). B) Selectivity experiments through comparison of the signal changes of random sequence, wild type, and mutant gene fragment using the perfect match sequence of crRNA (crRNA‐0). C) Illumination of the process for design 8 crRNAs with synthetic mismatches. D) The normalized results of 8 mismatch crRNA‐meditated detection assay for wild sequence and L858R mutation of *EGFR*. Error bars represent standard derivation obtained in three parallel experiments (*n* = 3).

In view of urgency for precisely profiling and monitoring ctDNA to direct therapeutic management in lung cancer therapy, our proposed strategy has been explored for the epidermal growth factor receptor (*EGFR*) L858R mutation, a potent predictive biomarker as ctDNA associated with lung cancer.^[^ ^37^ ^]^ The ctDNA has been selected to design the sequence of crRNA based on the requirements of protospacer adjacent motif (PAM) sequence (‐TTTN). The sequence of crRNA has been first designed by using wild type gene‐*EGFR* sequence. As shown in **Figure** **4**A, the requirements of PAM sequence (‐TTTN) are considered to design the perfect match sequence of crRNA (crRNA‐0) according to the substitution mutation (A>C for SNV in target sequence). As shown in Figure 4B, a much higher signal readout for SNV than the wild type can be recorded based on crRNA‐0‐medicated detection assay, however, there is still a relatively strong background signal, which is not conducive to detection.

In order to meet the detection requirements of higher signal‐to‐noise ratio, a mismatched primer is unitized to enable an efficient identification of SNV in mutated DNA sequences. As shown in Figure 4C, mismatches of adjacent bases for the point mutation were designed, in which a total of 8 crRNAs are synthesized to specifically recognize the sequence of L858R. The normalized results are shown in Figure 4D, with the base engineering of crRNA sequence, the activity of cas12a/crRNA from Mn‐MOFs has changed to different degrees. Clearly, it can be obtained thatCas12a/crRNA‐2 and Cas12a/crRNA‐3 remain high trans‐cleavage activity to sensing *EGFR* L858R mutation, but with a low signal response to wild type sequence. By calculating the signal ratio, crRNA‐2 sequence is determined to be the optimal crRNA. Certainly, MME‐CRISPR is inherently adaptable to diverse genetic targets by designing crRNAs complementary to mutation sites adjacent to compatible PAM sequences, as demonstrated by the successful detection of phosphatidylinositol 3‐kinase (*PIK3CA*) H1047R and Kirsten rat sarcoma viral oncogene (*KRAS*) A146T in Figure S16 (Supporting Information).

### 2.5 Analytical Performance of the Established Electrochemical Method

**Figure 5:**
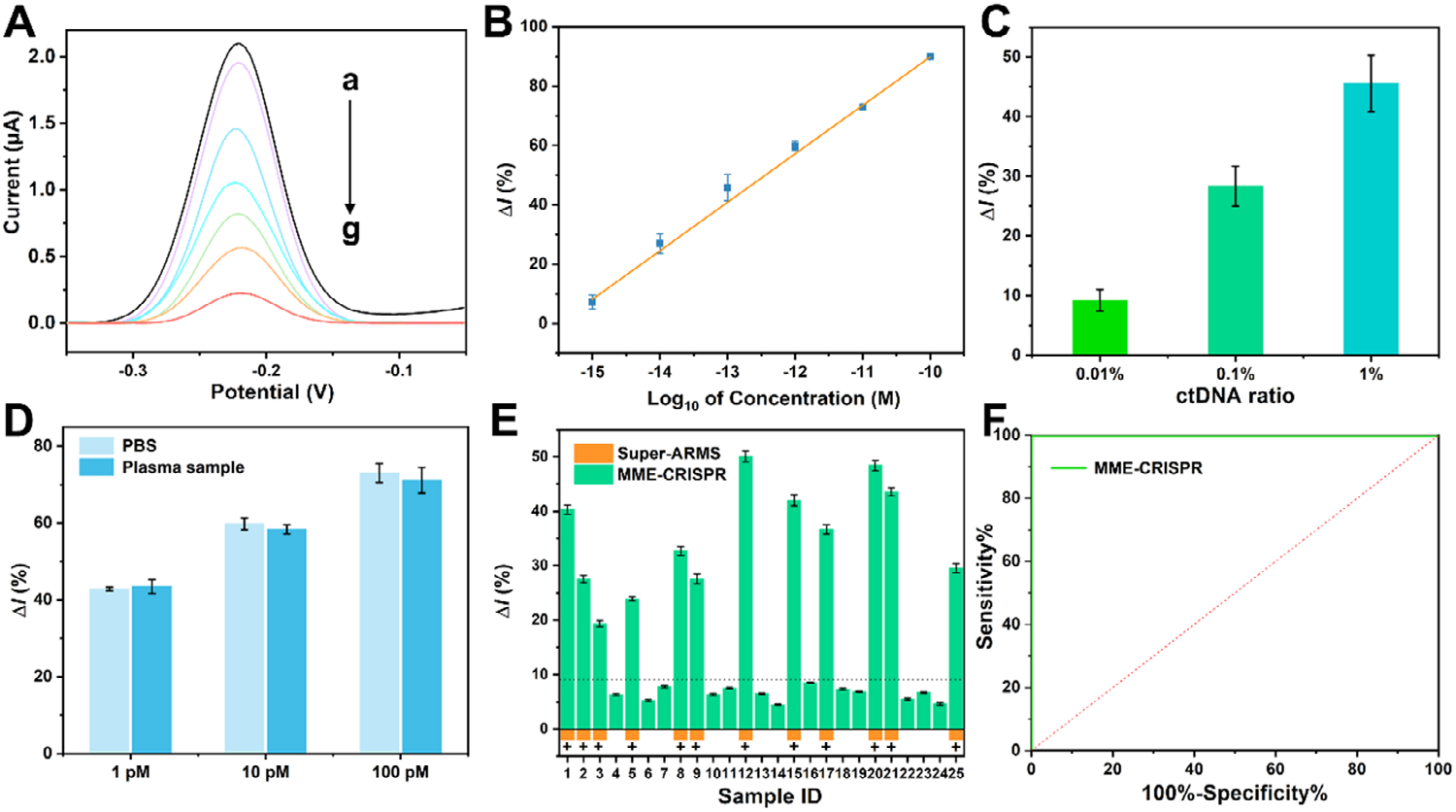
Analytical performance of the established electrochemical method. A) SWV response with different concentrations of target dsDNA. a–g, 1 fm, 10 fm, 100 fm, 1 pm, 10 pm, 100 pm, and 1 nm. B) The linear relationship between the SWV response and the logarithmic value of target dsDNA concentrations (1fm–100 pm). C) Results showing the detection performance of *EGFR* L858R at different mutation fractions from 0.01% to 1%. D) The current change obtained for target ctDNA in PBS and in human serum samples. E) The changed current signal of *EGFR* L858R detection in plasma samples using the biosensor based on MME‐CRISPR (Green column) and positive results identified by super‐ARMS method (Orange column) from 25 lung cancer patients. F) The corresponding ROC curve of this biosensor. Error bars represent standard derivation obtained in three parallel experiments (*n* = 3).

Under the optimized conditions including the ratio of Cas12a and crRNA of 2:1, MES buffer with pH 6, Mn^2+^ concentration of 10 mm, and incubation time of 15 min, different concentrations of target dsDNA has been detected by the developed electrochemical method. The results of SWV measurement are shown in **Figure** **5**A, and it can be found that the signal outputs gradually enhance with the increase of target concentrations. A linear relationship can be established between the change of current and logarithmic values of target dsDNA concentrations ranging from 1 fm to 100 pm. The regression equation is *△I* = 253.725 + 16.323lg*C* with a squared correlation coefficient of 0.998, where *△I* is the change of SWV response and *C* represents the concentration of target dsDNA (Figure 5B). The limit of detection is calculated to be as low as 0.28 fm, which is 2 orders of magnitude lower than that (33.2 fm) of using the free Mn^2+^ (Figure S17, Supporting Information). This assay also shows a better sensitivity on DNA detection compared with published works using electrochemical CRISPR system (Table S2, Supporting Information). The high sensitivity could be derived from the improved catalyzed efficiency of the enhanced CRISPR‐Cas12a system using Mn‐MOFs. Collectively, all the results indicate that this improved electrochemical biosensor could be used for efficient ctDNA sensing with good sensitivity and selectivity.

To validate the feasibility and clinical potential of our proposed method in practical application, the *EGFR* L858R mutation in real samples were detected. First, the mutation target in simulated sample has been prepared according to the proportion of ctDNA (from 0.01% to 1%) in plasma samples at different tumor stages. As shown in Figure 5C, this biosensor produces the response even at 0.01% ctDNA. In addition, the diluted healthy human serum samples spiked with different concentrations of target dsDNA were determined by this biosensor. As shown in Figure 5D, no statistical difference can be found between target dsDNA diluted into serum and that in buffer. The recovery ratios were calculated to be 97.38% to 102.1% with relative standard deviation (RSD) values ranging from 2.06% to 4.71% (Table S3, Supporting Information). Furthermore, plasma samples from 8 advanced lung cancer patients with clinically confirmed *EGFR* L858R mutation and 8 healthy individuals have been collected from a local hospital respectively to evaluate the potential for clinical application of this biosensor. Circulating DNA is enriched using VAHTSTM Serum/Plasma Circulating DNA Kit (Vazyme, China) for the test. As shown in Figure S18 (Supporting Information), the average value of the plasma samples from lung cancer patients is significantly larger than that of the healthy individuals.

To further validate the effectiveness, 25 samples have been collected from lung cancer patients and analyzed by MME‐CRISPR‐based biosensor and Super‐ARMS EGFR Mutation Detection Kit (AmoyDx, China). As shown in Figure 5E, the biosensor based on MME‐CRISPR can identify 12 L858R positives, which is consistent with Super‐ARMS method. Moreover, the corresponding the area under the curve (AUC) in receiver operating characteristic (ROC) curve of this biosensor is calculated to be 1 due to the high sensitivity and specificity (Figure 5F). These results demonstrate the established electrochemical biosensor based on MME‐CRISPR can perform assay of mutated DNA to benefit the diagnosis and monitoring progression of tumor.

## 3 Conclusion

In summary, we have first designed and constructed Cas12a/crRNA@Mn‐MOFs and realized the release of Cas12a/crRNA with the enhanced catalyzed ability stimulated by Mn^2+^ from metal‐organic framework in virtue of proximity effect. The coated Mn‐MOFs shell can provide great protection against the external environment. Meanwhile, benefiting from the tight interaction between Mn‐MOFs and Cas12a/crRNA, an amount of metal activators can be released from Mn‐MOFs under mild conditions to surround Cas12a proteins as stimulants. Therefore, the developed electrochemical biosensor based on MME‐CRISPR allows preamplification‐free detection for mutated ctDNA with ultra‐sensitivity. Meanwhile, the excellent targeting ability of Cas12a/crRNA using a mismatch crRNA sequence ensures the high specificity for point mutation detection. The proposed method has been used to analyze ctDNA mutation in clinical samples in a short time, providing a powerful tool for cancer diagnostic. Indeed, the developed strategy has some limitations such as the strict requirements of PAM sequences to analyze single nucleotide variants in ctDNA. However, we believe that this approach can be easily engineered to sensing a wide variety of nucleic acid targets by reasonably designing the structure of crRNA.

## 4 Experimental Section

### Materials and Reagents

Squaric acid and manganese (II) chloride tetrahydrate were ordered from Aladdin Chemistry Co., Ltd. (Shanghai, China). Engen Lba Cas12a (cpf1) proteins were purchased from NEW ENGLAND BioLabs Inc. (Beijing, China). AsCas12a, FnCas12a, and AacCas12b were obtained from Tolo Biotech Co., Ltd. (Shanghai, China). Tris‐(2‐carboxyethyl) phosphine hydrochloride (TCEP), 6‐mercaptohexanol (MCH), and bovine serum albumin (BSA) were ordered from Sigma–Aldrich Co., Ltd. (St. Louis, USA). All DNA and RNA sequences were synthesized and purified by Genscript Biotech Co., Ltd. (Nanjing, China). The sequences of the oligonucleotides are listed in Table S1 (Supporting Information). All the chemicals were of analytical grade without any further treatment. Deionized water was obtained from a Millipore water purification system (Milli‐Q, ≥18.2 MΩ) for the experiments.

Human plasma samples were obtained from the First Affiliated Hospital of Nanjing Medical University (Nanjing, China). Informed written consent from all participants was obtained prior to the research. All plasma sample studies were approved by the medical ethics committee of the First Affiliated Hospital of Nanjing Medical University (No. 2023‐SR‐277).

### Synthesis and Characterization of Mn‐MOFs and Cas12a/crRNA@Mn‐MOFs

182 mg squaric acid (1.6 mmol) and 128 mg NaOH (3.2 mmol) were dissolved in 16 mL water to prepare the squaric sodium solution (0.1 m). Then, 16 mL of 0.1 m manganese (II) chloride tetrahydrate solution containing 10% ethanol was added. After stirring for 10 min, the resulting products were collected by centrifugation at 10 000 rpm for 10 min and washed with deionized water three times. Finally, the precipitates were dried at 40 °C under reduced pressure overnight, to give Mn‐MOFs.

For the preparation of Cas12a/crRNA@Mn‐MOFs, Cas12a protein and crRNA were first incubated at room temperature for 15 min using a buffer prepared by nuclease free water containing 50 mm NaCl and 10 mm Tris‐HCl with a pH value of 7.4. Then 200 µL of 50 mm Mn^2+^ solution containing 10% ethanol was added to the prepared 200 µL Cas12a/crRNA solution (1 µm Cas12a and 0.5 µm crRNA), followed by the addition of 200 µL of 50 mm squaric sodium solution. After 10 min, the mixtures were centrifuged and washed with deionized water three times. Meanwhile, the supernatant solution was collected. Finally, the obtained precipitants were dried at 40 °C under reduced pressure overnight, to give Cas12a/crRNA@Mn‐MOFs.

The obtained Mn‐MOFs and Cas12a/crRNA@Mn‐MOFs were characterized by transmission electron microscope (TEM, H‐7650, Hitachi, Japan) and S‐3400N II scanning electron microscope (SEM, Hitachi, Japan), as well as IRPrestige 21 Fourier transform infrared spectrometer (FT‐IR, Shimadzu, Japan). Meanwhile, their X‐ray diffraction (XRD) data were collected by a D8 Advance diffractometer (Bruker, Germany). Additionally, the images of confocal laser scanning microscopy for Cas12a/FAM‐crRNA@Mn‐MOFs were recorded using LSM880 (Zeiss, Germany).

### Determination for the Encapsulation Efficiency of Cas12a/crRNA and Its Release from Mn‐MOFs

To determine the encapsulation efficiency of Cas12a/crRNA in Mn‐MOFs, FAM has been labeled at 3′ end of crRNA to prepare Cas12a/FAM‐crRNA@Mn‐MOFs. A standard curve was constructed for the free Cas12a/FAM‐crRNA through measuring the fluorescence intensity at 515 nm using an F‐7000 spectrometer (Hitachi, Japan). The efficiency can be calculated by recording the change of fluorescence intensity in supernatant solution after the assembly of Cas12a/crRNA@Mn‐MOFs.

Moreover, in order to study the process of Cas12a/FAM‐crRNA released from Mn‐MOFs, 200 µL MES buffer with different pH values (4, 5, 6, and 7) was utilized to monitor the fluorescence intensity of solution. After dissociation, a fresh Mn^2+^‐Cas12a/crRNA complex can be obtained, which can be further used for electrochemical measurements. Additionally, the concentration of Mn^2+^ bound to Cas12a/crRNA after ultrafiltration was determined by inductively coupled plasma optical emission spectroscopy (Thermo Scientific iCAP 7200 ICP‐OES, USA).

### Evaluation for Catalytic Efficiency of Cas12a/crRNA@Mn‐MOFs

First, target dsDNA (1 µm) was prepared by mixing target ssDNA and PAM sequence in hybridization buffer (10 mm Tris‐HCl, 50 mm NaCl, pH 7.4), heated to 95 °C for 5 min using a thermostatic metal bath (ThermoStat Plus, Eppendorf, Germany), and then immediately cooled at 4 °C for later use. To measure the changes of fluorescence intensities along with the reaction time obtained for Cas12a/crRNA@Mn‐MOFs, 10 µL of mixture containing the released Mn^2+^‐Cas12a/crRNA was mixed with 0.1 nm target dsDNA, 200 nm FAM/BHQ‐labeled single‐stranded DNA (FQ‐DNA), 50 mm NaCl, 10 mm Tris‐HCl, and 100 µg mL^−1^ BSA in a final volume of 50 µL. The intensity of fluorescence signal was recorded for 30 min using a multimode microplate reader (SPARK, TECAN, Swiss) with an excitation wavelength at 485 nm and emission wavelength at 535 nm. For the control experiments of Cas12a/crRNA with free divalent metal ions including Ca^2+^, Fe^2+^, Zn^2^⁺, Cu^2^⁺, Mg^2+^ or Mn^2+^, the prepared reaction buffer (50 mm NaCl, 10 mm Tris‐HCl, and 100 µg mL^−1^ BSA, pH 7.4) containing 10 mm corresponding metal ions were used.

For Michaelis‐Menten analysis, Cas12a/crRNA@Mn‐MOFs containing low concentrations of Cas12a/crRNA (10 nm Cas12a and 5 nm crRNA) was synthesized as described above. The prepared Cas12a/crRNA@Mn‐MOFs were mixed with 200 µL MES buffer (pH 6) and incubated for 5 min. Subsequently, 10 µL of mixture was added into a reaction buffer (10 nm target dsDNA, 50 mm NaCl, 10 mm Tris‐HCl, and 100 µg mL^−1^ BSA) containing different concentrations of FQ‐DNA (100, 200, 400, 800, 1000, and 2000 nm) in a final volume of 50 µL. The intensity of fluorescence signal was recorded for 600 s (free Mg^2+^) or 60 s (free Mn^2+^ and Cas12a/crRNA@Mn‐MOFs). Michaelis‐Menten equation was applied to calculate *k*_cat_ and *K*_m_ values.^[^ ^38^ ^]^

### Investigation for Stability of Cas12a/crRNA@Mn‐MOFs

*For heat treatment*: the Cas12a/crRNA@Mn‐MOFs particles and free Cas12a/crRNA (300 µL, 1 µm Cas12a, and 0.5 µm crRNA) solution were heated at 40, 60, or 80 °C for 30 min by a thermostatic metal bath (ThermoStat Plus, Eppendorf, Germany).

*For treatment by organic reagent*: Cas12a/crRNA@Mn‐MOFs particles were soaked in methanol or acetone (300 µL). After 1 h, they were centrifuged and washed with deionized water three times. Meanwhile, the free Cas12a/crRNA were added into acetone solvents (300 µL) in a final concentration of 1 µm Cas12a and 0.5 µm crRNA. After 1 h, they were filtered using a MWCO device (Millipore, 100 kDa).

*For treatment by proteolysis*: Cas12a/crRNA@Mn‐MOFs particles were soaked in solution containing 4 mg mL^−1^ proteolytic enzyme (protease K) for 1 h. Then, the mixture was centrifuged and washed with deionized water three times. Meanwhile, the free Cas12a/crRNA were added into the solution of proteolytic enzymes in a final concentration of 1 µm Cas12a and 0.5 µm crRNA. After 1 h, the mixture was filtered using a MWCO device (Millipore, 100 kDa).

After the treatments, Cas12a/crRNA@Mn‐MOFs were mixed with 200 µL MES buffer (pH 6) and incubated for 5 min. Subsequently, 10 µL of mixture was added into a reaction buffer (10 nm target dsDNA, 200 nm FQ‐DNA, 50 mm NaCl, 10 mm Tris‐HCl, and 100 µg mL^−1^ BSA) in a final volume of 50 µL. For the free Cas12a/crRNA, 10 mm of Mn^2+^ was added into the reaction buffer. Their catalytic activities were determined using a multimode microplate reader with an excitation wavelength at 485 nm and emission wavelength at 535 nm after 15 min.

### Preparation of MB‐DNA Modified Gold Electrode

Gold electrodes (3 mm in diameter) were cleaned according to the previously reported protocol.^[^ ^39^ ^]^ First, thiolated MB‐DNA (1 µm) was kept in 10 mm TCEP for 1 h. Then, 10 µL treated MB‐DNA was dropped onto the above cleaned gold electrode and incubated at 37 °C for 4 h in the dark. Subsequently, the resulting electrodes were dipped on 1 mm MCH solution for 2 h to form a highly aligned DNA monolayer. The prepared MB‐DNA modified electrodes were dried with nitrogen gas for the electrochemical experiments based on MME‐CRISPR.

### Electrochemical Measurement Based on MME‐CRISPR

Cas12a/crRNA@Mn‐MOFs was mixed with 200 µL MES buffer (pH 6), followed by incubation with different concentrations of target dsDNA or clinical samples in a reaction buffer containing 50 mm NaCl, 10 mm Tris‐HCl, and 100 µg mL^−1^ BSA (pH 7.4). Afterward, the resultant solution was dropped onto the electrode surface and incubated at 37 °C for 15 min. After washing, SWV measurements were achieved by using a CH Instruments 660D potentiostat (CH Instruments, Shanghai, China), with the scanning range from −0.1 to −0.6 V, an increment of 4 mV, an amplitude of 50 mV, and a frequency of 25 Hz. The electrochemical electrolyte was consisted of 20 mm PBS buffer (pH 7.4) and 0.5 m NaCl. Electrode impedance spectra (EIS) was measured in 0.1 m PBS (pH 7.4) containing 5 mm [Fe(CN)_6_]^3‐/4−^ and 1 m KCl.

### Statistical Analysis

The results presented are representative of at least three independent experiments and data were shown as mean ± standard deviation (S.D.). The sample size (n) for statistical analysis is included in figure legends. Data for designing crRNA were normalized to a range of between 0 and 1. For two groups comparisons, unpaired Student's t‐test was used. *p < 0.05 and **p < 0.01. Statistical analysis was carried out with GraphPad Prism 8.0 and Origin 2020 software.

## Conflict of Interest

The authors declare no conflict of interest.

## Supporting information



Supporting Information

## Data Availability

The data that support the findings of this study are available from the corresponding author upon reasonable request.
