# Supplementary material for: Enhanced the Trans‐Cleavage Activity of CRISPR‐Cas12a Using Metal‐Organic Frameworks as Stimulants for Efficient Electrochemical Sensing of Circulating Tumor DNA
Source: Adv Sci (Weinh). 2025 Apr 4;12(22):2417206. doi: 10.1002/advs.202417206 (PMC12165068; doi:10.1002/advs.202417206)
Supplement: Supplementary file 1 — Supporting Information [file ADVS-12-2417206-s001.docx]

**Supporting Information**

**Enhanced** **the** **Trans-Cleavage Activity of CRISPR-Cas12a using Metal-Organic Frameworks** **as Stimulants for** **Efficient Electrochemical Sensing of Circulating Tumor DNA**

Shuai Wu ^a^, Yincheng Liu ^b^, Tianyu Zeng ^c^, Tianci Zhou ^d^, Yanting Sun ^c^, Ying Deng ^d^, Juan Zhang ^e^ **^*^**, Genxi Li ^d, e^ **^*^**, Yongmei Yin ^c^ **^*^**

*^a^**Clinical Research Center, the First Affiliated Hospital with Nanjing Medical University, Nanjing, Jiangsu, 210029, P. R. China.*

*^b^Department of Breast Disease, the First Affiliated Hospital of Nanjing Medical University, Nanjing 210029, P. R. China*

*^c^Department of Oncology, the First Affiliated Hospital of Nanjing Medical University, Nanjing 210029, P. R. China.*

*^d^State Key Laboratory of Analytical Chemistry for Life Science, School of Life Sciences, Nanjing University, Nanjing 210023, P. R. China*

*^e^Center for Molecular Recognition and Biosensing, School of Life Sciences,* *Shanghai University, Shanghai 200444, P. R. China.*

* Corresponding authors.

E-mail addresses: juanzhang@shu.edu.cn (J. Zhang); genxili@nju.edu.cn (G. Li); ymyin@njmu.edu.cn (Y. Yin)





**Fig. S1.** Fluorescent intensity of the reaction solution containing 200 nM Cas12a, 100 nM crRNA, 200 nM FQ-DNA, and different concentration activator dsDNA (10 nM and 0.1 nM) in reaction buffer (50 mM NaCl, 10 mM Tris-HCl, 100 µg/ml BSA, 10 mM corresponding metal ions, pH 7.4) after 15 mins using a multimode microplate reader (SPARK, TECAN, Swiss) with an excitation wavelength at 485 nm and emission wavelength at 535 nm. Data represent mean ± S.D. (n = 3).


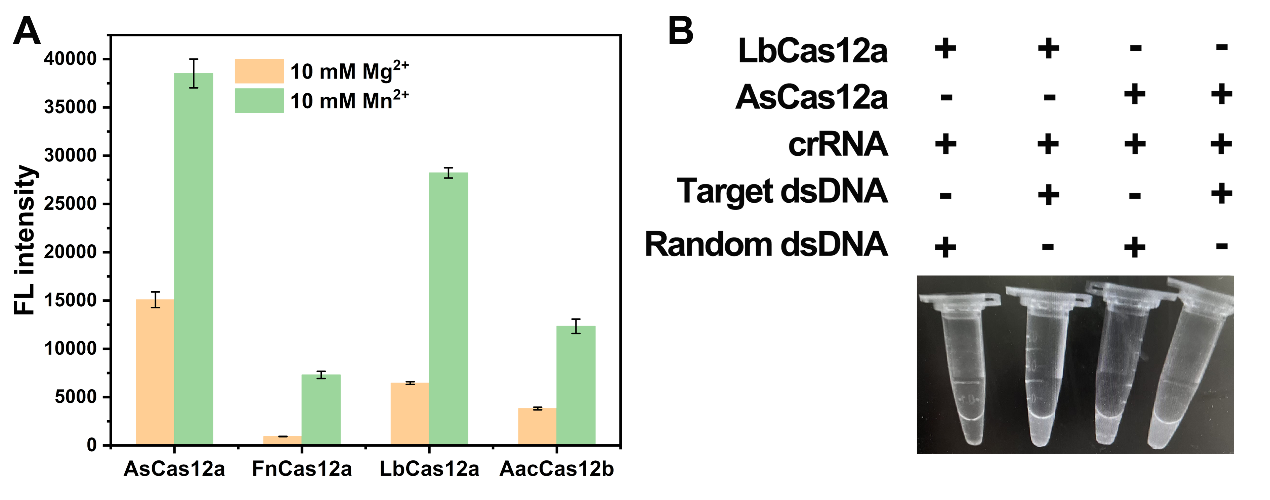


**Fig. S2.** (A) Fluorescent intensity of the reaction solution containing 100 nM crRNA or sgRNA, 200 nM FQ-DNA, 10 nM activator dsDNA, and 200 nM Cas12 proteins (AsCas12a, FnCas12a, LbCas12a, and AacCas12b) in reaction buffer (50 mM NaCl, 10 mM Tris-HCl, 100 µg/ml BSA, 10 mM Mg^2+^ and Mn^2+^, pH 7.4) after 15 mins using a multimode microplate reader (SPARK, TECAN, Swiss) with an excitation wavelength at 485 nm and emission wavelength at 535 nm. (B) The fluorescent image of AsCas12a and LbCas12a for fluorescent quenched ssDNA cleavage after 15 mins using a Biorad ChemiDoc XRS^+^ gel imaging system. Data represent mean ± S.D. (n = 3).





**Fig. S3.** Plot of FL intensity at 515 nm versus varying concentrations of free Cas12a/FAM-crRNA with the ratio of 2:1, where the horizontal coordinate is the protein concentration from 0.05 to 1 µM. Data represent mean ± S.D. (n = 3).





**Fig. S4.** Fluorescent spectra of the reaction solution containing 200 nM Cas12a, 100 nM crRNA, and 200 nM FQ-DNA in 1×NEBuffer™ r2.1 (50 mM NaCl, 10 mM Tris-HCl, 10 mM MgCl_2_,100 µg/ml BSA, pH 7.9), and different concentration activator dsDNA (0, 25, and 100 nM) after 15 mins using an F-7000 spectrometer.



 **Fig. S5.** The changes of fluorescence intensities along with the reaction time obtained for free Cas12a/crRNA Mn^2+^ (pH 7.4 and pH 6.0) in the presence of 0.1 nM target dsDNA. Data represent mean ± S.D. (n = 3).





**Fig. S6.** The linear correlation between the fluorescence intensity and the concentration of the completely cleaved reporter substrate. The fluorescence gain parameter of the instrument was adjusted to 20% to prevent signal saturation. Data represent mean ± S.D. (n = 3).


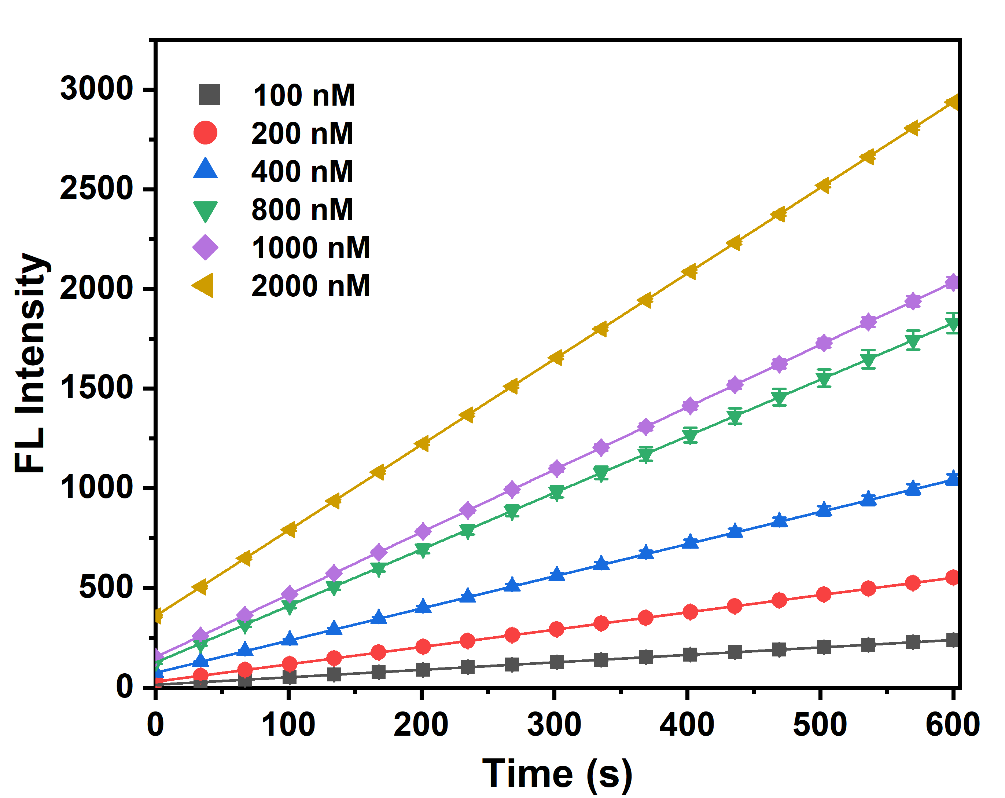


**Fig. S7.** Raw measurements of fluorescence signal associated with trans-cleavage kinetics experiments for Cas12a/crRNA with free Mg^2+^. Fluorescence intensity versus time (the first 600 s) for varying concentration of reporters between 100 nM and 2000 nM, and for 1 nM of Cas12a/crRNA activated by target dsDNA. Data represent mean ± S.D. (n = 3).





**Fig. S8.** Raw measurements of fluorescence signal associated with trans-cleavage kinetics experiments for Cas12a/crRNA with free Mn^2+^. Fluorescence intensity versus time (the first 60 s) for varying concentration of reporters between 100 nM and 2000 nM, and for 1 nM of Cas12a/crRNA activated by target dsDNA. Data represent mean ± S.D. (n = 3).





**Fig. S9.** Raw measurements of fluorescence signal associated with trans-cleavage kinetics experiments for Cas12a/crRNA@Mn-MOFs. Fluorescence intensity versus time (the first 60 s) for varying concentration of reporters between 100 nM and 2000 nM, and for 1 nM of Cas12a/crRNA activated by target dsDNA. Data represent mean ± S.D. (n = 3).


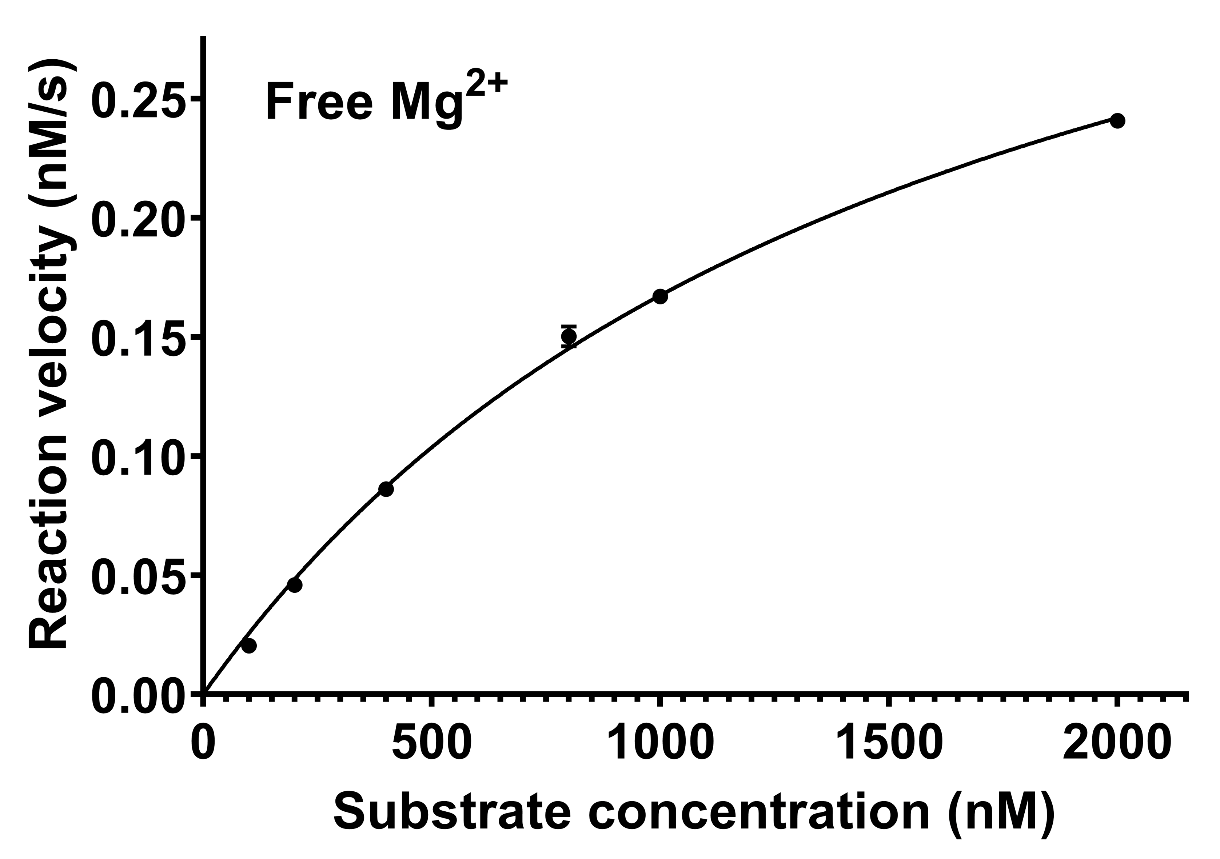


**Fig. S10.** A clearer version of Michaelis-Menten plot for the cleavage activity of free Cas12a/crRNA with Mg^2+^. Data represent mean ± S.D. (n = 3).


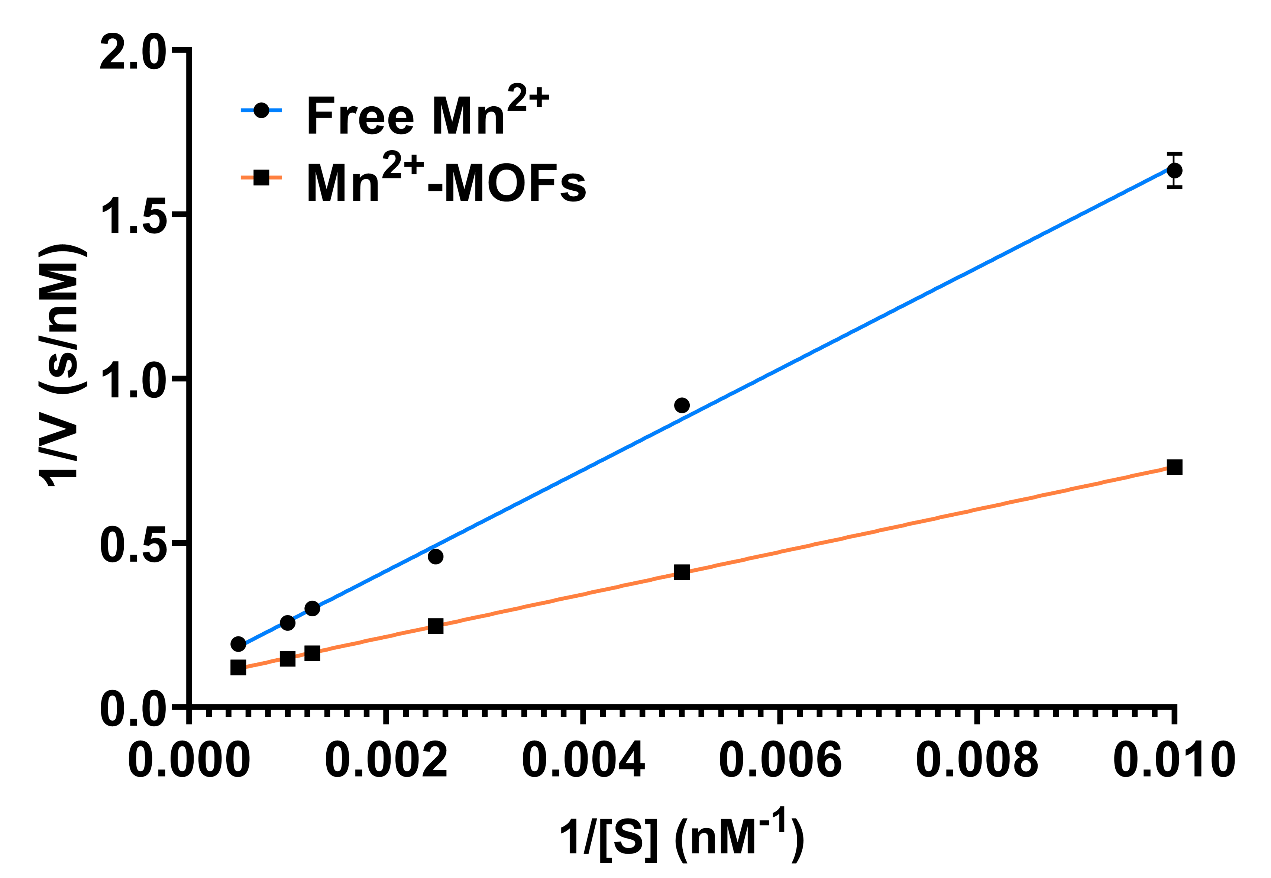


**Fig. S11.** A clearer version of Lineweaver-Burk plot for the cleavage activity of free Cas12a/crRNA with free Mn^2+^ and Cas12a/crRNA@Mn-MOFs. Data represent mean ± S.D. (n = 3).





**Fig. S12.** The concentration of Mn bound to the released Cas12a/crRNA tracked by ICP-OES after ultrafiltration at different time. Data represent mean ± S.D. (n = 3).





**Fig. S13.** Protective performance of Mn-MOFs for Cas12a/crRNA in the long-term storage test. Data represent mean ± S.D. (n = 3).





**Fig. S14.** EIS curves of the electrode in 0.1 M PBS (pH=7.4) containing 5 mM [Fe(CN)_6_]^3-/4-^ and 1 M KCl. (a) bare gold electrode, (b) MB-DNA/Au, (c) MME-CRISPR/MB-DNA/Au.





**Fig. S15.** Square wave voltammograms of MB-DNA modified gold electrodes with different pH (pH 4, 5, and 6).


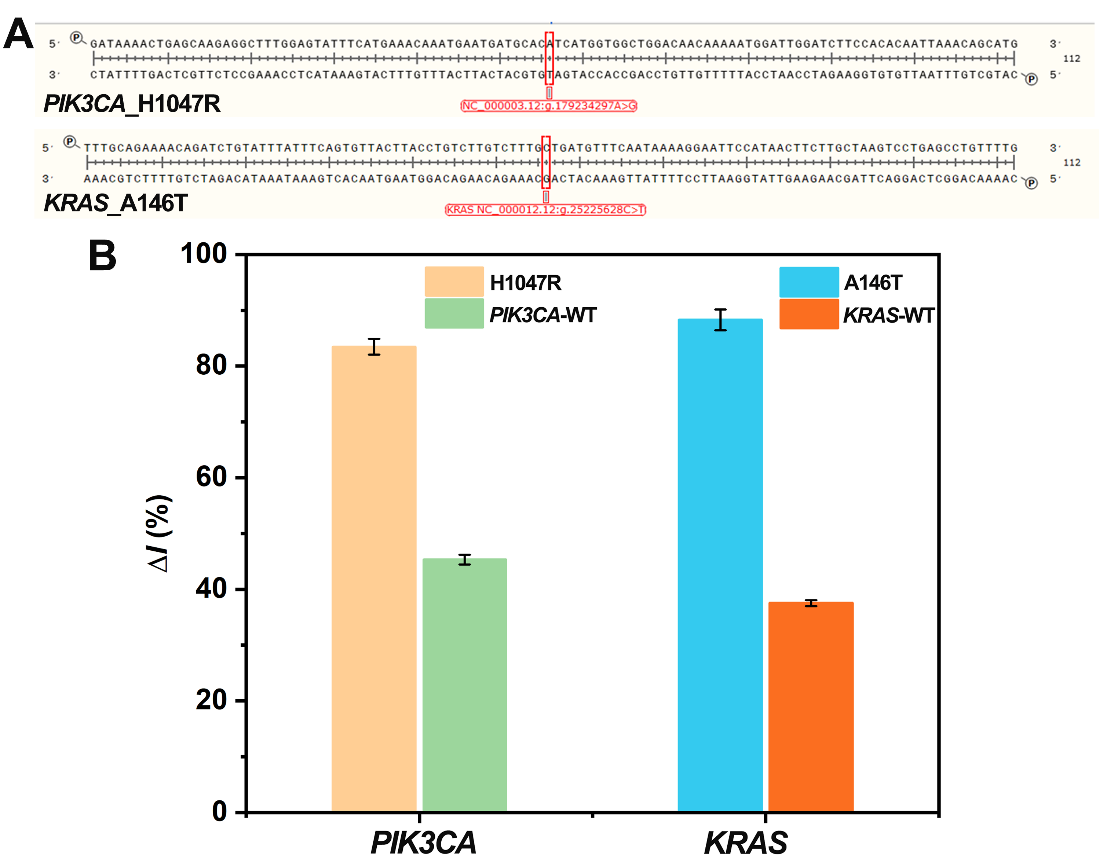


**Fig. S16.** (A) Schematic of the H1047R mutation between the wild type and mutant (A-to-G) in the fragment of gene *PIK3CA* and the A146T mutation between the wild type and mutant (C-to-T) in the fragment of gene *KRAS*. (B) The comparison of the signal changes of 0.1 nM wild type and related mutant gene fragment (*PIK3CA*_H104R and *KRAS*_A146T). Data represent mean ± S.D. (n = 3).





**Fig. S17.** The linear relationship between the SWV response and the logarithm of target DNA concentration (100 fM-10 nM) with free Mn^2+^. The regression equation is Y=133.562+9.546lgC with a squared correlation coefficient of 0.996. Error bars represent standard derivation obtained in three parallel experiments (n=3).





**Fig. S18.** The changed current signal of *EGFR* L858R detection in plasma samples from 8 healthy people and 8 advanced lung cancer patients. Error bars represent standard derivation obtained in three parallel experiments (n=3).

**Table S1.** DNA and RNA sequences employed in this work

| Name | Sequences |
| --- | --- |
| CrRNA-0  CrRNA-1  CrRNA-2  CrRNA-3  CrRNA-4  CrRNA-5  CrRNA-6  CrRNA-7  CrRNA-8  FAM-CrRNA  L858R-Target  Wild-Target  Random Target  L858R-PAM  Wild-PAM  Random-PAM  MB-DNA  FQ-DNA  AacCas12b-sgRNA  H1047R-crRNA  H1047R-Target  H1047R-PAM  *PIK3CA*WT-Target  *PIK3CA*WT-PAM  A146T-crRNA  A146T-Target  A146T-PAM  *KRAS*WT-Target  *KRAS*WT-PAM | 5’-UAAUUUCUACUAAGUGUAGAUGGCGGGCCAAACUGCUGGG  UGCG-3’  5’-UAAUUUCUACUAAGUGUAGAUGGAGGGCCAAACUGCUGGG  UGCG-3’  5’-UAAUUUCUACUAAGUGUAGAUGGGGGGCCAAACUGCUGGG  UGCG-3’  5’-UAAUUUCUACUAAGUGUAGAUGGUGGGCCAAACUGCUGGG  UGCG-3’  5’-UAAUUUCUACUAAGUGUAGAUGGCGAGCCAAACUGCUGGG  UGCG-3’  5’-UAAUUUCUACUAAGUGUAGAUGGCGCGCCAAACUGCUGGG  UGCG-3’  5’-UAAUUUCUACUAAGUGUAGAUGGCGUGCCAAACUGCUGGG  UGCG-3’  5’-UAAUUUCUACUAAGUGUAGAUGGCGGUCCAAACUGCUGGG  UGCG-3’  5’-UAAUUUCUACUAAGUGUAGAUGGCGGUCGAAACUGCUGGG  UGCG-3’  5’-UAAUUUCUACUAAGUGUAGAUGGCGGGCCAAACUGCUGGG  UGCG-/3’-FAM/  5’-CGCACCCAGCAGTTTGGCCCGCCCAAAATCTGTGATCTTG  ACA-3’  5’-CGCACCCAGCAGTTTGGCCAGCCCAAAATCTGTGATCTTG  AC-3’  5’-GACTGCGCATCATGGTGCGGACGTGCAAAGACAAAATGGAT  TGGA-3’  5’-TGTCAAGATCACAGATTTTGGGCGGGCCAAACTGCTGGGT  GCG-3’  5’-TGTCAAGATCACAGATTTTGGGTGGGCCAAACTGCTGGGT  GCG-3’  5’-TCCAATCCATTTTGTCTTTGCACGTCCGCACCATGATGCGCA  GTC-3’  /5’-Thiol Modifier C6 S-S/-AAAAAAAAAAAAAAAA-/3’ MB/  /5’-FAM/-TTTATT-/3’-BHQ/  5’-GUCUAGAGGACAGAAUUUUUCAACGGGUGUGCCAAUGGC  CACUUUCCAGGUGGCAAAGCCCGUUGAGCUUCUCAAAUCUG  AGAAGUGGCACGGCGGGCCAAACUGCUGGGUGCG-3’  5’-UAAUUUCUACUAAGUGUAGAUUUGUCCAGCCACCAUGACG  UGCA-3’  5’-TGCACGTCATGGTGGCTGGACAACAAA-3’  5’-TTTGTTGTCCAGCCACCATGACGTGCA-3’  5’-TGCACATCATGGTGGCTGGACAACAAA-3’  5’-TTTGTTGTCCAGCCACCATGAGGTGCA-3’  5’-UAAUUUCUACUAAGUGUAGAUUUGAUGUUUCAAUAAAAG  GAAUU-3’  5’- AATTCCTTTTATTGAAACATCAACAAA-3’  5’-TTTGTTGATGTTTCAATAAAAGGAATT-3’  5’- AATTCCTTTTATTGAAACATCAGCAAA-3’  5’-TTTGCTGATGTTTCAATAAAAGGAATT-3’ |

**Table S2.** Comparison of Electrochemical CRISPR system for nucleic acid detection

|  | LOD | Refs |
| --- | --- | --- |
| Cas12a-based universal electrochemical biosensor  Cas13a-based electrochemical microfluidic biosensor  Cas12a-based electrochemiluminescence biosensor  Cas12a-based DNA framework-supported biosensor  Cas12a-based biosensor utilizing silver metallization  Mn-MOFs enhanced Cas12a-based biosensor | 50 pM  10 pM  0.48 pM  100 fM  3.5 fM  0.28 fM | ^[1]^  ^[2]^  ^[3]^  ^[4]^  ^[5]^  This method |

**Table S3.** Detection of target DNA in real samples

| Sample | Added (pM) | Detected (pM) | Recovery (%) | RSD (%) |
| --- | --- | --- | --- | --- |
| 1  2  3 | 1  10  100 | 1.021  9.762  97.38 | 102.1  97.62  97.38 | 4.16%  2.06%  4.71% |

**References**

[1] Y. Dai, R. A. Somoza, L. Wang, J. F. Welter, Y. Li, A. I. Caplan, C. C. Liu, *Angew. Chem.-Int. Ed.* **2019**, *58*, 17399–17405.

[2] R. Bruch, J. Baaske, C. Chatelle, M. Meirich, S. Madlener, W. Weber, C. Dincer, G. A. Urban, *Adv. Mater.* **2019**, *31*, 1905311.

[3] P.-F. Liu, K.-R. Zhao, Z.-J. Liu, L. Wang, S.-Y. Ye, G.-X. Liang, *Biosens. Bioelectron.* **2021**, *176*, 112954.

[4] J. Su, Y. Ke, N. Maboyi, X. Zhi, S. Yan, F. Li, B. Zhao, X. Jia, S. Song, X. Ding, *Small Methods* **2021**, 2100935.

[5] A. Suea-Ngam, P. D. Howes, A. J. deMello, *Chem. Sci.* **2021**, *12*, 12733–12743.
